# Transcriptional patterns reveal tumor histologic heterogeneity and immunotherapy response in lung adenocarcinoma

**DOI:** 10.3389/fimmu.2022.957751

**Published:** 2022-08-08

**Authors:** Mengxue Jiao, Hui Liu, Xuejun Liu

**Affiliations:** School of Computer Science and Technology, Nanjing Tech University, Nanjing, China

**Keywords:** intratumor heterogeneity, immune microenvironment, histologic progression, prognostic model, transcriptional pattern

## Abstract

Tumoral heterogeneity has proven to be a leading cause of difference in prognosis and acquired drug resistance. High intratumor heterogeneity often means poor clinical response and prognosis. Histopathological subtypes suggest tumor heterogeneity evolved during the progression of lung adenocarcinoma, but the exploration of its molecular mechanisms remains limited. In this work, we first verified that transcriptional patterns of a set of differentially expressed genes profoundly revealed the histologic progression of lung adenocarcinoma. Next, a predictive model based on the transcriptional patterns was established to accurately distinguish histologic subtypes. Two crucial genes were identified and used to construct a tumor heterogeneous scoring model (L2SITH) to stratify patients, and we found that patients with low heterogeneity score had better prognosis. Low L2SITH scores implied low tumor purity and beneficial tumor microenvironment. Moreover, L2SITH effectively identified cohorts with better responses to anti–PD-1 immunotherapy.

## 1 Introduction

Non–small cell lung cancer (NSCLC) is one of the malignant tumors over the world (1), and its morbidity and mortality increased gradually in recent years. The 5-year survival rate of patients with NSCLC is only 18%. Lung adenocarcinoma (LUAD) is the main subtype, accounting for 40% of patients with NSCLC (2). LUAD has distinctive histological stages during its progression. Pathologists have categorized difference of histological phenotype, referred to as histologic subtypes, including lepidic, papillary, acinar, and solid (3). With the histologic progression from lepidic to solid, LUADs become increasingly aggressive and metastatic.

The immune system was supposed to drive intertumor and intratumor heterogeneity by exerting different selective pressures to different regions of the solid tumor (4). Prior studies have shown significant difference of stroma and immune infiltrating cells in different intratumor regions, as well as interpatient tumors (5, 6). Accordingly, the tumor microenvironment has been shown to play an important role in tumor growth, angiogenesis, immune evasion, and metastasis (7). Substantial evidence suggests that tumor heterogeneity increases the likelihood that cancer cells survive conventional chemotherapy and targeted anticancer drugs (8–10). In addition, tumor heterogeneity affects the efficacy of immunotherapies, especially immune checkpoint inhibitors (11–13). However, the exploration of molecular mechanism underlying the histologic heterogeneity remained nascent (14).

In fact, the potential molecular mechanism of histologic heterogeneity of LUAD is multifaceted. The genomic aberrations, epigenetic modifications, small-molecule RNA, malfunction of transcriptional regulations, and environmental factors may lead to phenotypic differences (15–17). Studies have found strong associations between the histologic heterogeneity and prognosis of LUAD, but the investigation of molecular signature underlying each histologic subtype is scarce (18). In this paper, we set about to find molecular determinations of tumor heterogeneity from the perspective of histologic subtypes. The immunogenetic transcriptional patterns showed strong link to the histologic progression and tumor microenvironment. We established a heterogeneous scoring model (L2SITH) based on the molecular signatures to stratify patients and found that patients in the low-scored group had better prognosis, which was more predictive than the stratification based on histologic subtypes. In contrast, histologic subtypes did not showed significant prognostic value for patients with LUAD. Moreover, L2SITH effectively identified cohorts with better responses to anti–PD-1 immunotherapy.

## 2 Materials and methods

### 2.1 Data sources

Two LUAD cohorts from The Cancer Genome Atlas (TCGA) (N = 246) and Gene Expression Omnibus (GEO) (GSE58772, N = 48) were included in our study. The RNA-seq and clinical data were obtained from the Genomic Data Commons (https://gdc.cancer.gov/) and GEO database (https://www.ncbi.nlm. nih.gov/geo/), respectively.

Intratumoral heterogeneity of these samples was annotated as lepidic (L), papillary (P), acinar (A), or solid (S) subtypes, according to the most popular histopathological classification standard (3). The annotations of the TCGA cohort were from (19), which included lepidic (N = 10), papillary (N = 50), acinar (N = 69), and solid (N = 58) patients. The GEO GSE58772 cohort included lepidic (N = 10), papillary (N = 18), acinar (N = 10). and solid (N = 10) samples (20).

The immune subtypes of 455 LUAD samples were marked by Thorsson et al. (21), including C1 (wound healing, N = 82), C2 (Interferon (IFN)-dominant, N = 147), C3 (inflammatory, N = 178), C4 (lymphocyte depleted, N = 20), and C6 (Transforming Growth Factor (TGF)-dominant, N = 28).

The cohort treated with the PD-1 inhibitor Nivolumab was obtained from GEO (GSE126044, N = 16). This cohort included 11 non-responders (11 PD cases) and five responders (one SD case and four partial response (PR) cases).

### 2.2 Differential expression and enrichment analysis

The *DESeq2* R package (22) was used to conduct differential analysis between normal and two most representative subtypes, including lepidic vs. normal, solid vs. normal, and lepidic vs. solid. The differentially expressed genes were chosen using the filtering criterion of absolute ǀ *log*_2_ *FoldChange* ǀ > 1 and p.adj < 0.05. The differential expression genes overlapped with immune-related genes from InnateDB were filtered out for further analysis. Out of the 1,952 immune genes, we got 96 immune-related differentially expressed genes.

The *clusterProfiler* R package (23) was applied for Gene Ontology (GO) functional annotation and Kyoto Encyclopedia of Genes and Genomes (KEGG) pathway enrichment analysis. The *GOPlot* R package (24) was used to calculate the z-score with the filtering threshold of P.adj < 0.05 to select statistically significant pathways.

### 2.3 Histologic subtype clustering analysis

To visualize the molecular signature difference among histologic subtypes, ComplexHeatmap package (25) was used to draw the heatmap of expression profiles. The *tSNE* (26) and *UMAP* (27) tools were used to perform dimensionality reduction and clustering of expression profile of genes underlying the tumor heterogeneity. The *ggplot2* package (28) was used to display the clusters.

### 2.4 MLP model for histologic subtype classification

A multilayer perceptron (MLP) model was constructed using the *Neuralnet* (29) package. The input layer included 38 nodes corresponding to differentially expressed immune-related genes. The two hidden layers include 11 and 9 nodes, respectively. There were three nodes in the output layer for classification of histologic subtypes. The performance of the classification model was evaluated by ROC curve and Receiver Operating Characteristic (ROC)-Area Under Curve (AUC) values. The sklearn and matplotlib tools were used to calculate the ROC-AUC values and plot ROC curves.

### 2.5 Random forest for immune subtype classification

The transcriptomic data of filtered genes were transformed to *z*-scores and then fed into a random forest model to predict immune subtypes (C1, C2, C3, C4, or C6). The samples were split to training and test set by 7:3 ratio. The random forest model included 50 decision trees with a maximum tree depth of 5 and a maximum number of leaf nodes of 50. The sklearn and matplotlib tools were used to calculate the ROC-AUC values and plot ROC curves.

### 2.6 Tumor purity and immune microenvironment analysis

The *ESTIMATE* (30) tool was used to calculate the tumor purity, stromal, and immune scores. The *t*-test was used for statistical significance. The *corrplot* (31) package was used to plot heatmap, in which *p* < 0.05 was denoted by *, *p* < 0.01 by **, and *p* < 0.001 by ***.

### 2.7 Establishment of L2SITH score model

We performed univariate and multivariate Cox regression analysis regarding the set of genes related to histologic subtype. The genes significantly related to prognosis were used to construct the heterogeneity score model L2SITH. The *survival* tepallison2010survival package was used to run survival analysis for high- and low-scored group, and the Kaplan-Meier (K-M) curves were plotted by *survminer* (32) package. The pRRophetic (33) package was used for drug proposal based on Cancer Cell Line Encyclopedia drug sensitivity dataset.

## 3 Results

### 3.1 Transcriptional patterns reflect histologic progression

**Figure 1:**
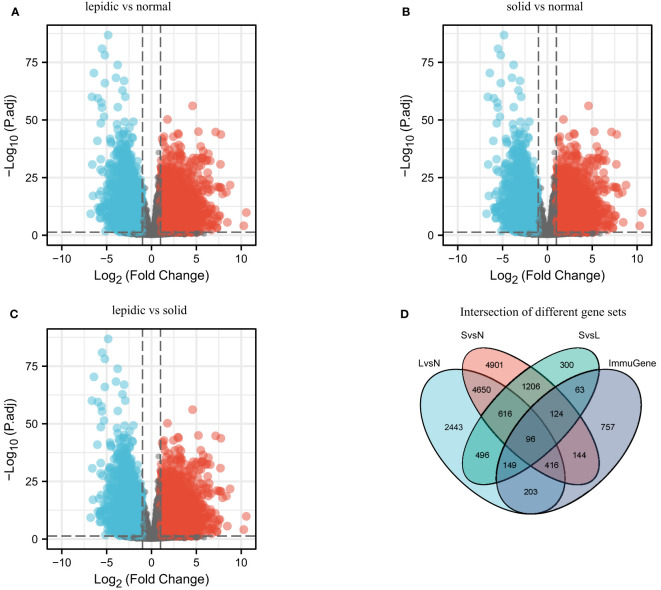
Differential analysis between normal, lepidic, and solid histologic subtypes. **(A)** Differential expression genes between lepidic and normal samples (LvsN). **(B)** Differential expression genes between solid and normal samples (SvsN). **(C)** Differential expression genes between lepidic and solid samples (LvsS). **(D)** Intersection of differentially expressed genes and immune genes (ImmuGene).

**Figure 2:**
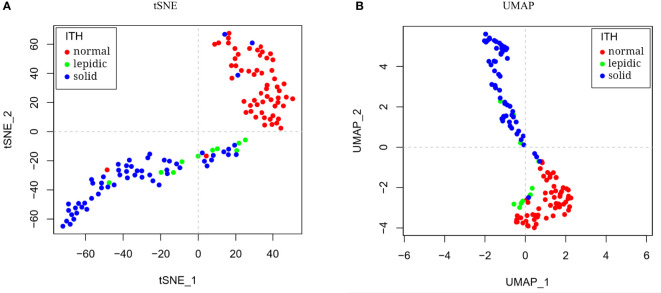
Dimension reduction and visualization of histologic subtypes. The LUAD samples showed separate distribution by **(A)** tSNE and **(B)** UMAP dimension reduction on transcriptional profiles.

Prior studies have reported the histologic heterogeneity of LUAD, which mainly included four subtypes: lepidic, papillary, acinar, and solid during tumor progression (3). From lepidic to solid, tumor aggressiveness and metastasis increase. For simplicity, we mainly focused on lepidic and solid patterns, regarding papillary and acinar as intermediate state. On the basis of the differential expression analysis between normal, lepidic, and solid samples, 96 differentially expressed genes related to immunologic function were filtered out, as shown in **Figure 1**. After dimensionality reduction and visualization by tSNE and UMAP tools, the transcriptional patterns clearly distinguished the histologic subtypes, as shown in **Figure 2**. The enriched GO annotations of 96 differentially expressed immune genes were shown in **Figure S1**. The 38 significantly upregulated immune genes showed positive correlation (**Figures S2**, **S3**).

**Figure 3:**
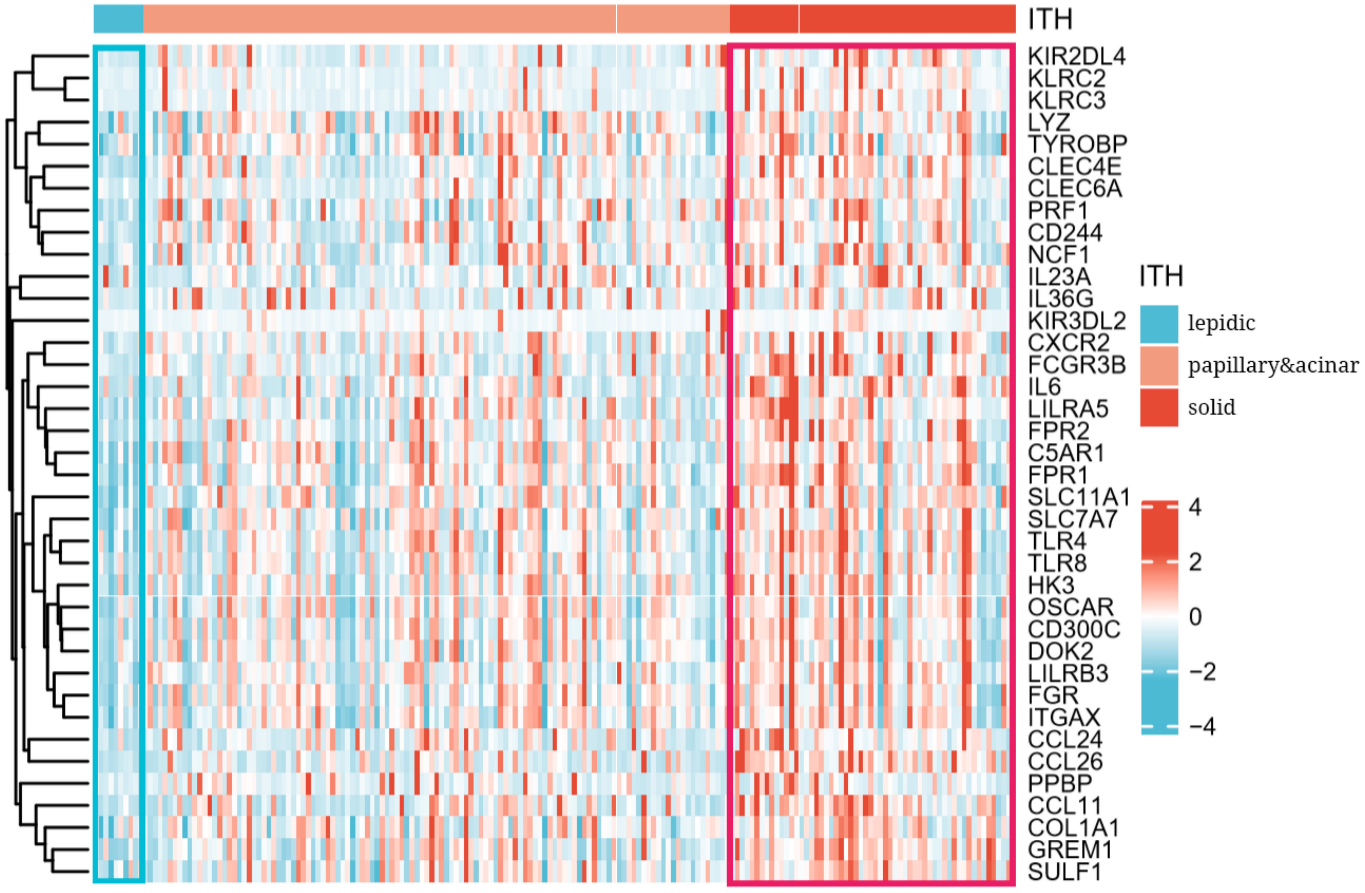
Heatmap of transcriptional patterns reflected histologic progression from lepidic to solid subtype.

As shown in **Figure 3**, these screened genes showed distinctive transcriptional patterns among different histologic subtypes. Interestingly, their expression levels increased significantly from lepidic to solid pattern. This may implied the activation of the immune response during tumor progression so that more and more immune cells infiltrated into the tumor. Our analysis preliminarily verified that transcriptional patterns of immunologic genes reflected the histologic heterogeneity in LUAD.

### 3.2 Transcriptional patterns accurately predict histologic subtypes

**Figure 4:**
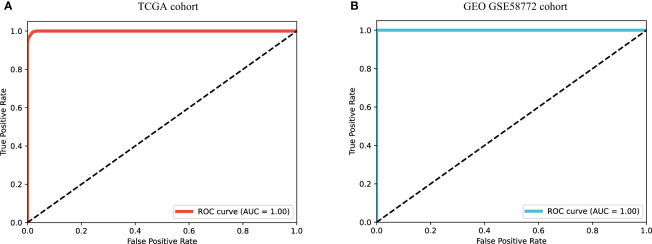
RCO curves of MLP model for histologic subtype prediction using transcriptional pattern. **(A)** ROC curve on TCGA cohort. **(B)** ROC curve on GEO GSE58772 cohort.

As molecular mechanism underlies the cell phenotype, we supposed transcriptional patterns should be predictive of phenotypic label. For this purpose, a MLP model was trained to classify histologic subtypes. We split the 187 samples into three types according to histologic subtypes: lepidic (N = 10), papillary and acinar (N = 119), and solid (N = 58). This was a typical multi-class classification task, based on the molecular signature of the set of immune-related genes. Expectedly, the MLP model achieved extremely high performance. The ROC-AUC was close to 1, and the accuracy rate reaches 97%, as shown in **Figure 4A**. In particular, 8 of 10 lepidic samples were correctly classified, 117 of 119 papillary and acinar samples were correctly classified, and all 58 solid samples were correctly classified. Moreover, we found that, as the histologic pattern progressed, a higher accuracy of the model is achieved. This implied that progressive tumor tended to develop distinctive molecular signatures that dominate the histological morphology and cell phenotype.

To verify the generalization, we verified the classification model on a GEO cohort (N = 48, where L = 10, P&A = 28, and S = 10). We were pleased to find that the model can completely distinguish the three types of histologic subtypes correctly, as shown in **Figure 4B**. The independent test set fully validated the outstanding potential of immunogenetic molecular signature in differentiating tumor histologic heterogeneity.

### 3.3 Establishment of heterogeneity scoring model L2SITH

**Figure 5:**
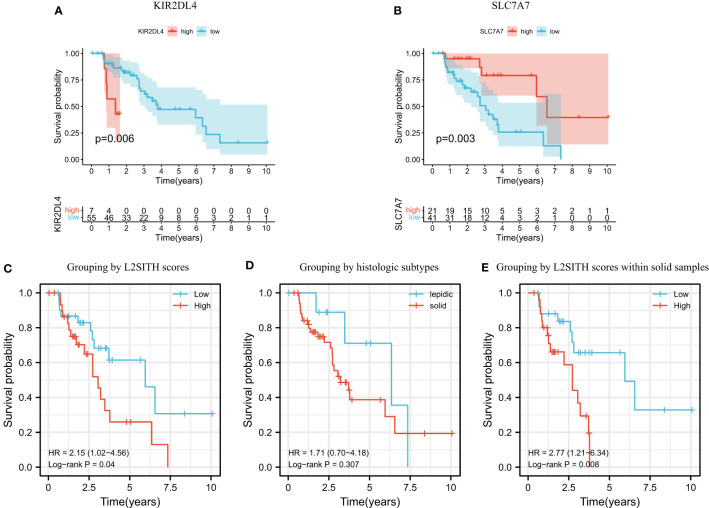
Survival analysis of two key genes, L2SITH heterogeneity score, and histologic phenotype. Panels **(A, B)** showed the K-M survival curves of patients grouped by KIR2DL4 and SLC7A7 gene, respectively. **(C)** K-M survival curve of patients grouped by L2SITH heterogeneity score. **(D)** K-M survival curve of patients grouped by lepidic and solid subtypes. **(E)** K-M survival curve of the solid group patients divided by L2SITH heterogeneity score.

We further performed Cox regression and survival analysis to screen genes significantly related to prognosis and obtained two key genes KIR2DL4 and SLC7A7. It has been reported that KIR2DL4 was highly associated with cancer development (34), and its lower expression level means better prognosis (**Figure 5A**). SLC7A7 was a suppressor gene inhibiting the progression of LUAD (35), and its higher expression level yielded to better prognosis (**Figure 5B**). Therefore, we established the heterogeneity score model L2SITH using these two genes, using the regression coefficients and the transcriptional levels of these two genes. Its formal definition is L2SITH = −0.0003708 * SLC7A7 (express) + 0.0047614 * KIR2DL4 (express). Using the L2SITH score, we divided the 62 lepidic and solid patients into high- and low-scored groups. The KM survival curves showed that the low-scored group had significantly high overall survival rate, as shown in **Figure 5C**. However, if the patients were divided into groups by histologic subtype (lepidic vs. solid), then the overall survival has no significant difference (**Figure 5D**). This suggested that the L2SITH model captured molecular factors underlying tumor progression, thereby acquired better prognostic power than the histologic heterogeneity. To verify this point, we further divided the solid samples (N = 52) using L2SITH scores and showed that the low-scored subgroup still had better overall survival, as shown in **Figure 5E**.

### 3.4 L2SITH revealed beneficial tumor microenvironment

**Figure 6:**
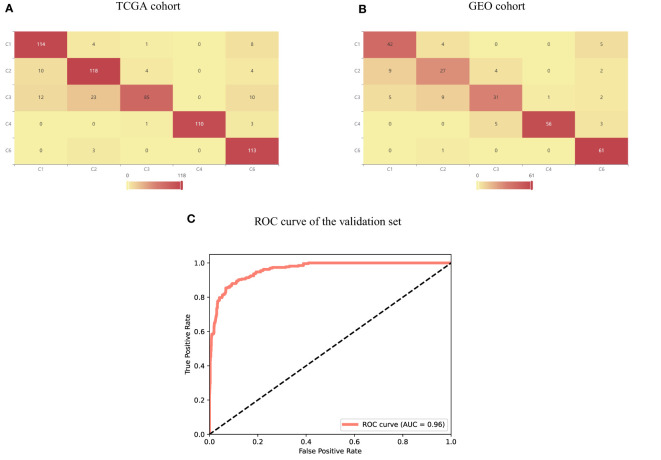
Performance of immune subtypes prediction based on transcriptional pattern. **(A)** Immune subtype prediction results of TCGA cohort. **(B)** Immune subtype prediction results of GEO cohort. **(C)** ROC curve of immune subtype prediction on GEO cohort.

To validate the association between transcriptional patterns and tumor microenvironment, we adopted the immune subtypes established by Thorsson et al. (21) for analysis. We divided the 890 samples by 7:3 for training and test and built a random forest model to predict the immune subtypes from molecular signature. On the training and test set, the accuracy reached 0.867 and 0.813, respectively. The detail of each immune subtypes was shown in **Figures 6A, B**, and the ROC curve was shown in **Figure 6C**. This demonstrated that the transcriptional patterns of the key immune-related genes can significantly differentiate immune subtypes of LUAD.

**Figure 7:**
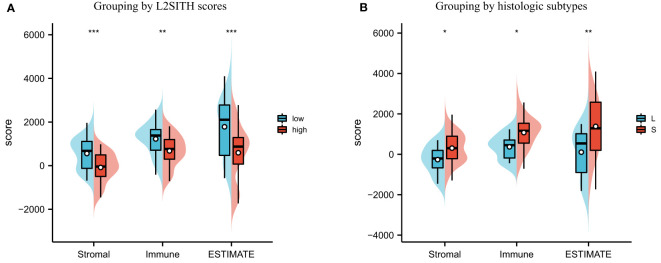
Tumor purity estimation of LUAD samples grouped by L2SITH score and histologic subtype. **(A)** Stromal, immune, and ESTIMATE scores of high- and low-scored groups. **(B)** Stromal, immune, and ESTIMATE scores of lepidic and solid histologic subtypes. The symbol * indicates statistical significance at the 0.05 level (p<0.05), ** indicates the level of significance 0.01 (p<0.01), *** indicates the level of significance 0.001 (p<0.001).

We used ESTIMATE tool to evaluate the immune infiltration level of the high- and low-scored group, to explore the significance of the L2SITH in the stratification of tumor microenvironment. As shown in **Figure 7**, we found that low-scored L2SITH group had higher stromal level, immune infiltration, and ESTIMATE scores than high-scored group. Conversely, when samples were grouped by histologic subtype, we observed a different trend, that is, the solid samples had higher stromal level, immune infiltration, and ESTIMATE scores than lepidic samples. The results indicated that the patients with low L2SITH scores had low tumor purity and beneficial tumor microenvironment to immunotherapy.

### 3.5 L2SITH accurately predicts clinical immunotherapy response

**Figure 8:**
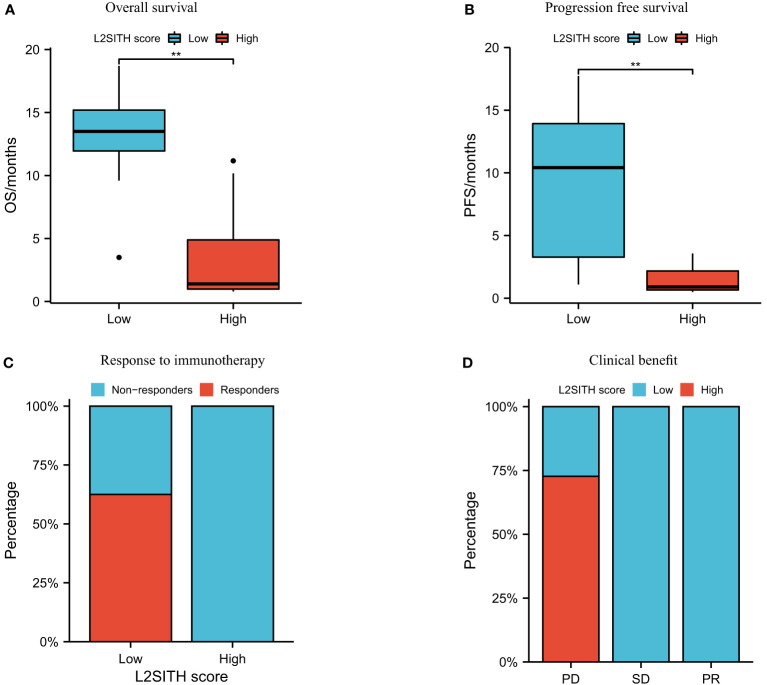
Survival and clinical response to anti–PD-1 inhibitor (Nivolumab) of high- and low-L2SITH score groups. **(A)** Overall survival of patients in high- and low-scored groups. **(B)** Progression-free survival of patients in high- and low-scored groups. **(C)** Percentage of patients with response to Nivolumab high- and low-scored groups. **(D)** Percentage of high- and low-scored patients with clinical response from Nivolumab, including progressive disease (PD), stable disease (SD), and partial response (PR). The symbol ** indicates significant difference at the 0.01 level (p<0.01).

To validate the effectiveness of the L2SITH score model in predicting response to immunotherapy, we analyzed a cohort of patients with NSCLC received Nivolumab PD-1 inhibitor treatment. Using the L2SITH to divide the sample into high- and low-scored groups, we observed significant difference in clinical response (**Figures 8A, B**). Clearly, the low-scored group had notably better OS and PFS than high-scored group. Furthermore, we found that the patients who responded to Nivolumab treatment were all in the low-scored group, as shown in **Figure 8C**. The clinical response to immunotherapy in the high-scored group was weak, and these patients suffered progressive disease (**Figure 8D**). This confirmed the reliable performance of L2SITH in predicting the immune response of NSCLC.

**Figure 9:**
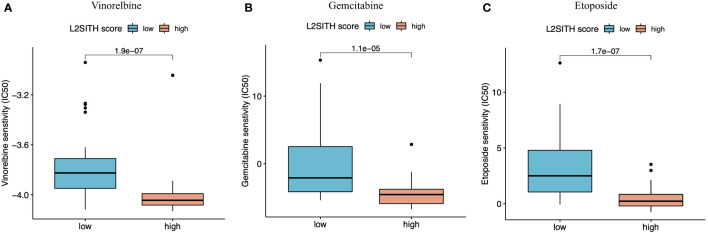
Potential beneficial drugs to patients with LUAD grouped by L2SITH score. **(A)** Vinorelbine, **(B)** gemcitabine, and **(C)** etoposide were inferred to benefit the low-scored LUAD patients.

In addition to the prognostic value, we further utilized the L2SITH scores to screen potential beneficial drugs. Three approved drugs, vinorelbine, gemcitabine, and etoposide, were predicted to have higher potential sensitivity to the low-scored group stratified by L2SITH (**Figure 9**). Vinorelbine is an anti-mitotic chemotherapy drug that was approved in 1990s for the treatment of non-NSCLC (29). According to a recent study, vinorelbine is a suitable choice for elderly patients with NSCLC and also a partner drug with immunochemotherapy (36). Gemcitabine is a nucleoside metabolism inhibitor approved by FDA in combination with cisplatin for the treatment of NSCLC (37). Low-dose gemcitabine treatment is sufficient to inhibit tumor growth with few side effects *in vivo*. Gemcitabine can also activate antitumor immune response in patients with normal immune system (38). Etoposide, a coccine toxin derivative, has also been shown to be useful in the treatment of small cell lung tumors (39). Etoposide plus cisplatin chemotherapy improved the efficacy and safety of small cell lung cancer (40).

## 4 Discussion

At present, the prognosis of patients with cancer depends mainly on the clinical staging and pathological grading. In recent years, more and more studies have shown that intratumor heterogeneity is an important factor of clinical treatment efficacy and prognosis. Tumors with high heterogeneity tend to be more aggressive and indicate poor prognosis. In fact, interpatient and intratumor heterogeneity is prevalent in both lymphoma and solid tumor. Apart from the molecular feature difference, the heterogeneity is also reflected in immune microenvironment, such as different immune infiltration level and tumor purity.

This study tried to reveal the underlying molecular features of histologic heterogeneity in LUAD. Through in-depth exploration of the transcriptional profiles of immune-related genes, we constructed a machine learning model to predict histologic subtypes. In addition, the transcriptional profiles were highly predictive of tumor immune subtypes. From these results, the molecular signature reflects the essential causes of phenotypic differences.

We found significant correlation between the transcriptional pattern of immune genes and the histologic progression (**Figure S4**). During the early stage, most immune genes were not activated and showed low transcriptional levels. With the histologic progression, immune genes were activated along with the tumor immune infiltration increased. This was reflected by the significant positive correlation between the expression of immune genes and histologic subtypes from lepidic to solid. We also explored the correlation of the differentially expressed immune genes with mutation type, but no significant association was found (**Figure S5**).

From the perspective of histologic progression, the lepidic cells were more differentiated, whereas solid cells were poorly differentiated and aggressive, but we did not observed significant difference in overall survival between lepidic and solid groups. This motivated us to find the molecular factors that really lead to differences in histologic subtypes and survival. Upon the transcriptional profiles, we developed L2SITH, a simple but predictive two-gene score model. The patient stratified by L2SITH scores showed statistically significant differences in overall survival. Within the solid group, L2SITH score model was still effective in distinguishing patients with better prognosis. On the other hand, this suggested that patients grouped in the same histologic subtypes still have significantly different molecular mechanisms. Rather than phenotypic differences, molecular mechanisms are the real reasons for tumor progression. In fact, a prior study (14) has explored the impact of epigenetic factors on histological heterogeneity. It concluded that the LUAD histological progression from lepidic to solid was mainly caused by epigenetic and transcriptional factors. We were exactly inspired by this prior study and focused on the transcriptomic mechanism that actually drove the histological progression but has not been explored before. In addition, we are also interested in the small-molecule RNAs that are potentially associated to histological subtypes and plan to explore such association analysis in the near future work.

Finally, we tried to explore the association of genomic mutations with the histologic progression in LUAD. However, the mutation landscape of driver genes cannot reveal the histologic progression. Moreover, we inspected the most highly mutated genes in LUAD but found no consistent trends of histologic progression driven by highly mutated gene (**Figures S6**, **S7**). These were consistent with the conclusion that the histologic progression is not dominated by genetic mutations (14). Further, we explored the relationship between L2SITH grouping and genomic mutations. The samples within each histologic subtype were split into high- and low-scored subgroups, and the mutations of each subgroup were shown in **Figures S8–** **S10**. Although our L2SITH model cannot distinguish the mutations, we found that, as the sample size increased, the number of genes with significant differences between high- and low-scored subgroups increased.

## Data availability statement

The datasets presented in this study can be found in online repositories. The names of the repository/repositories and accession number(s) can be found in the article/**Supplementary Material**.

## Author contributions

MJ and HL conceived the main idea and the framework of the manuscript. MJ performed the experiments. XL and HL helped to improve the idea. MJ drafted the manuscript and HL revised the manuscript. HL supervised the study and provided funding. All authors read and commented on the manuscript.

## Funding

This work was supported by the National Natural Science Foundation of China under grant no. 62072058.

## Conflict of interests

The authors declare that the research was conducted in the absence of any commercial or financial relationships that could be construed as a potential conflict of interest.

## Publisher’s note

All claims expressed in this article are solely those of the authors and do not necessarily represent those of their affiliated organizations, or those of the publisher, the editors and the reviewers. Any product that may be evaluated in this article, or claim that may be made by its manufacturer, is not guaranteed or endorsed by the publisher.
