# Supplementary material for: Transcriptional patterns reveal tumor histologic heterogeneity and immunotherapy response in lung adenocarcinoma
Source: Front Immunol. 2022 Aug 8;13:957751. doi: 10.3389/fimmu.2022.957751 (PMC9393366; doi:10.3389/fimmu.2022.957751)
Supplement: Supplementary file 1 [file DataSheet_1.pdf]

Supplementary Material

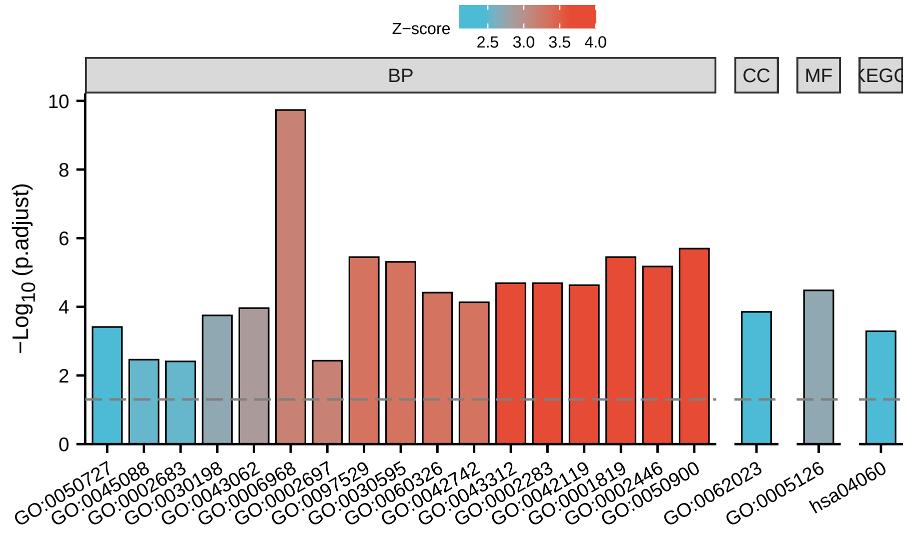

Figure S1. GO and KEGG pathway enrichment analysis on 96 differentially expressed immune-related genes

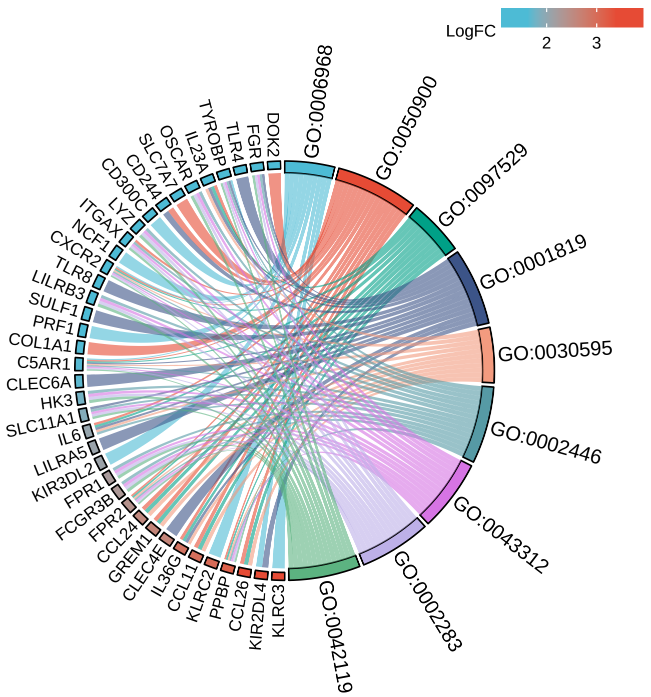

Figure S2. Top 9 GO functional annotations with most biological and the associations with 38 differentially expressed immune-related genes. It was found that the majority of these genes were up-regulated.

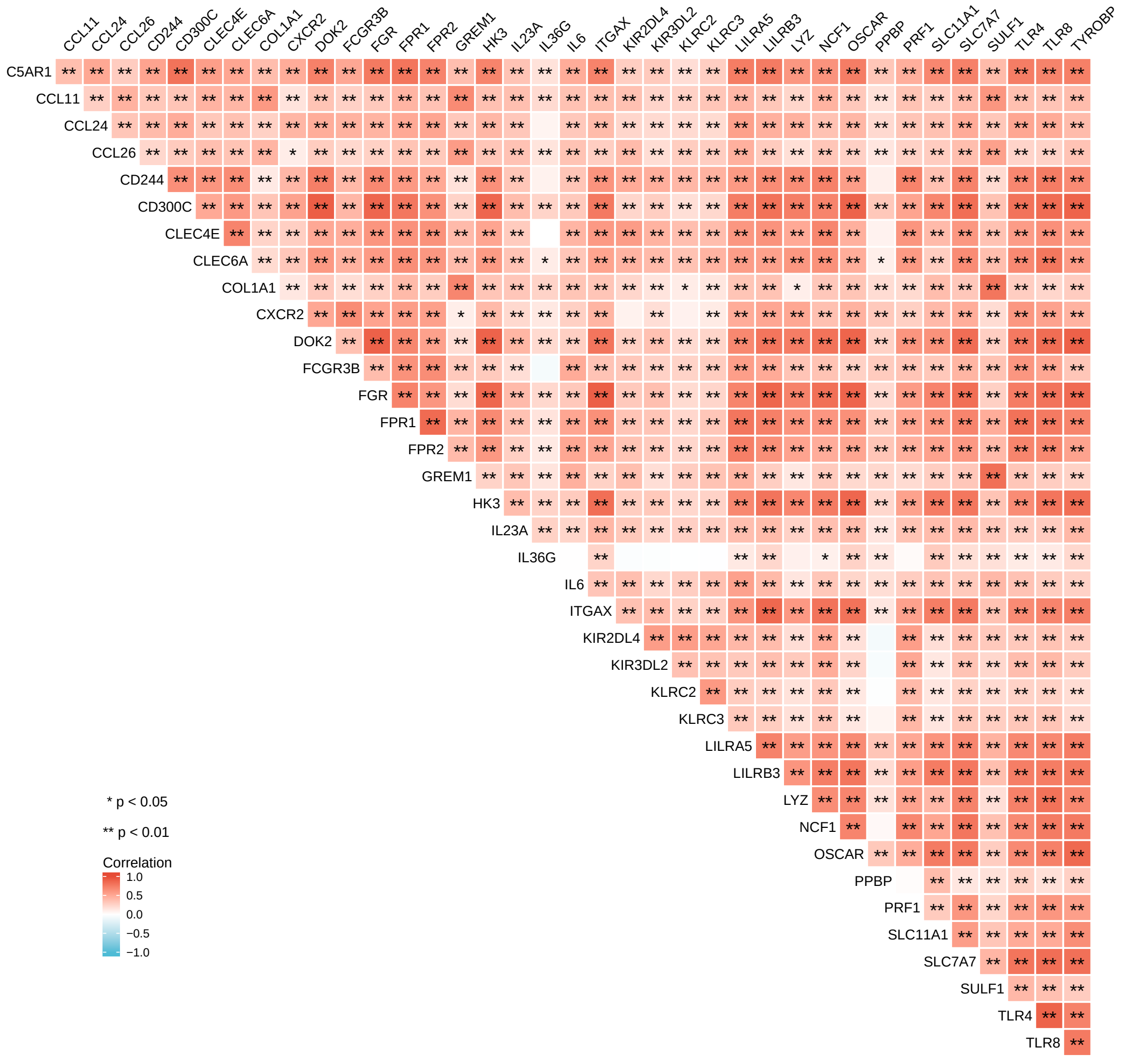

Figure S3. Pearson correlation matrix of 38 differentially expressed immune-related genes. Most gene pairs showed significantly positive correlation of expression profiles.

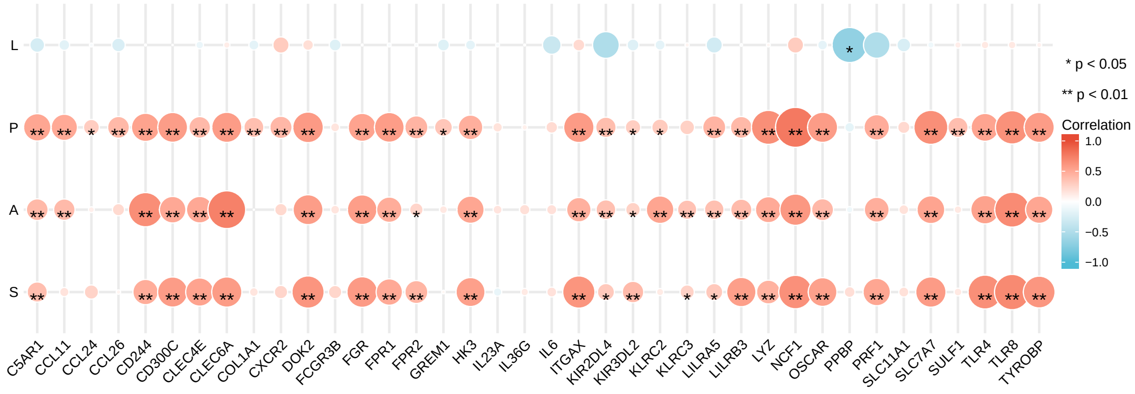

Figure S4. The correlation between 38 differentially expressed immune-related genes and immune histological patterns. In the early histological pattern was very low, but as the histological pattern progressed, more and more immune cells infiltrated into the tumor, and the content of immune cells gradually increased. Also, the expression of immune genes was positively correlated with the progression of the histological pattern.

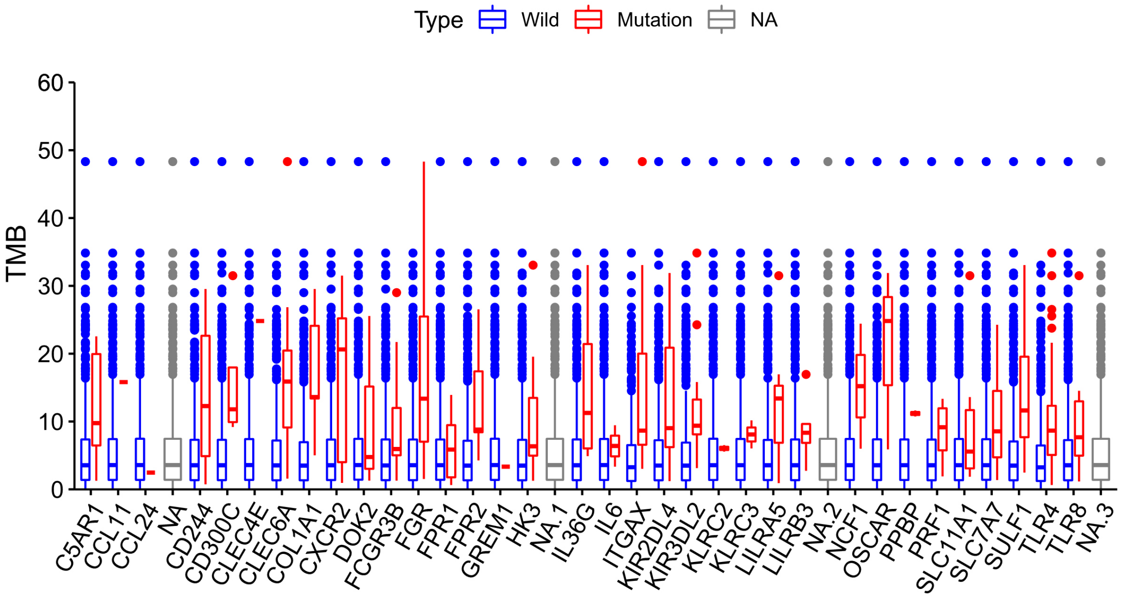

Figure S5. Genomic variations of 38 differentially expressed immune-related genes in wildtype and tumor tissues.

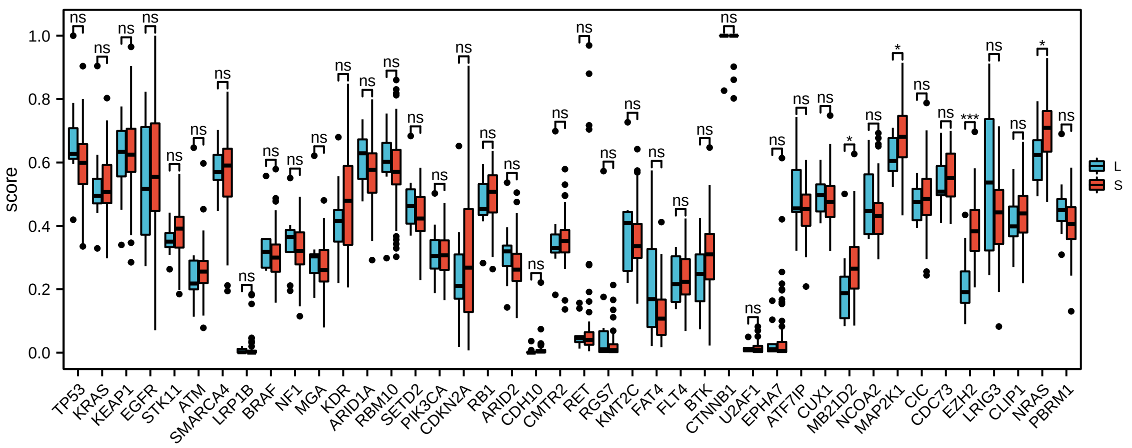

Figure S6. Transcriptomic level of LUAD cancer driver genes in lepidic and solid histological subtypes. There was no significant associations between driver gene expression patterns and histological progression

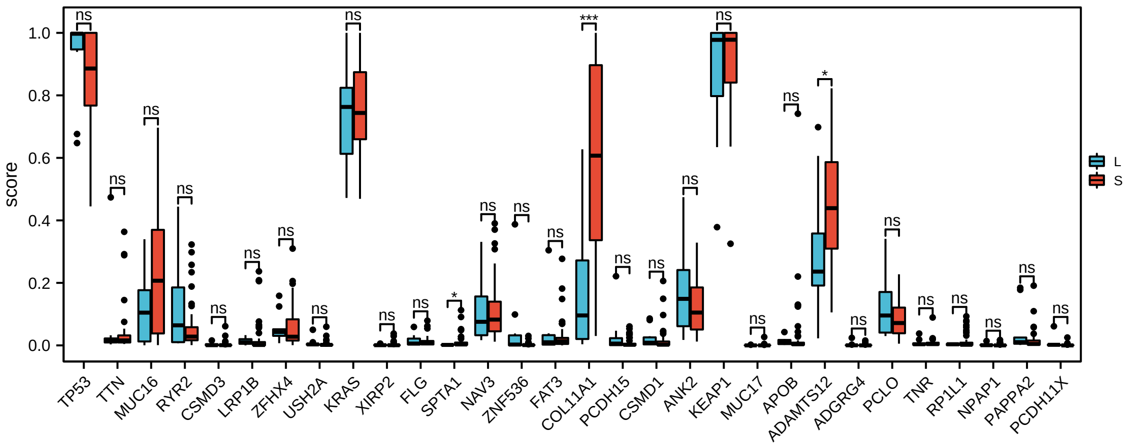

Figure S7. Transcriptomic levels of top 30 genes with the highest mutation frequencies and histological progression. There was no significant associations between most-mutated gene expression patterns and histological progression

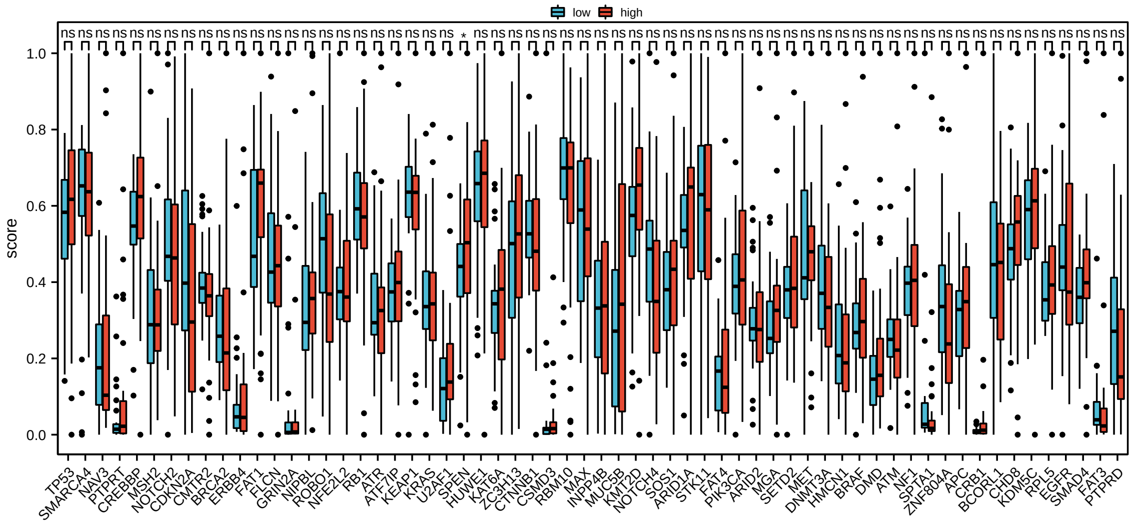

Figure S8. Expression levels of cancer driver genes in L2SITH-stratified samples within lepidic and solid subtypes. No significant difference of cancer driver gene expression levels between high and low subgroups was found.

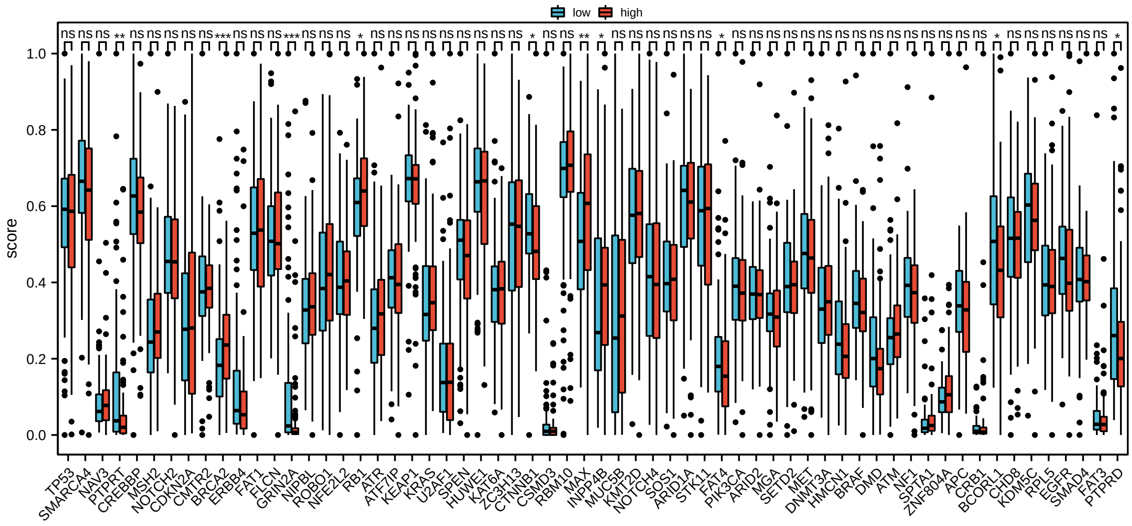

Figure S9. Expression levels of mostly mutated genes between L2SITH-stratified samples. No significant difference of cancer driver gene expression levels between high and low subgroups was found. However, the number of significantly mutated genes increased with histological progression.

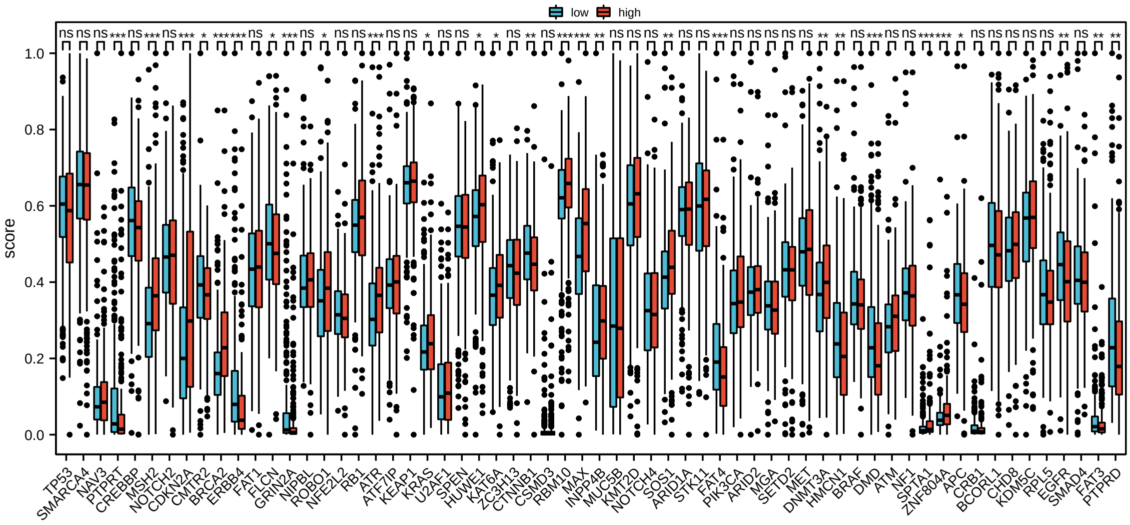

Figure S10. Expression levels of mostly mutated genes between L2SITH-stratified samples in TCGA LUAD cohort. It can be found that the number of differential genes between the high- and low- risk groups divided by L2SITH increased, when the sample size enlarged.
